# Complementary and Alternative Medicine for Cancer Pain: An Overview of Systematic Reviews

**DOI:** 10.1155/2014/170396

**Published:** 2014-04-13

**Authors:** Yanju Bao, Xiangying Kong, Liping Yang, Rui Liu, Zhan Shi, Weidong Li, Baojin Hua, Wei Hou

**Affiliations:** ^1^Department of Oncology, Guang'anmen Hospital, China Academy of Chinese Medical Sciences, Beixiange 5, Xicheng District, Beijing 100053, China; ^2^Institute of Chinese Materia Medica, China Academy of Chinese Medical Sciences, Nanxiaojie, Dongzhimennei Avenue, Beijing 100700, China; ^3^Department of Nephrology, Guang'anmen Hospital, China Academy of Chinese Medical Sciences, Beixiange 5, Xicheng District, Beijing 100053, China

## Abstract

*Background and Objective*. Now with more and more published systematic reviews of Complementary and Alternative Medicine (CAM) on adult cancer pain, it is necessary to use the methods of overview of systematic review to summarize available evidence, appraise the evidence level, and give suggestions to future research and practice. *Methods*. A comprehensive search (the Cochrane Library, PubMed, Embase, and ISI Web of Knowledge) was conducted to identify all systematic reviews or meta-analyses of CAM on adult cancer pain. And the evidence levels were evaluated using GRADE approach. *Results*. 27 systematic reviews were included. Based on available evidence, we could find that psychoeducational interventions, music interventions, acupuncture plus drug therapy, Chinese herbal medicine plus cancer therapy, compound kushen injection, reflexology, lycopene, TENS, qigong, cupping, cannabis, Reiki, homeopathy (Traumeel), and creative arts therapies might have beneficial effects on adult cancer pain. No benefits were found for acupuncture (versus drug therapy or shame acupuncture), and the results were inconsistent for massage therapy, transcutaneous electric nerve stimulation (TENS), and *Viscum album* L plus cancer treatment. However, the evidence levels for these interventions were low or moderate due to high risk of bias and/or small sample size of primary studies. *Conclusion*. CAM may be beneficial for alleviating cancer pain, but the evidence levels were found to be low or moderate. Future large and rigor randomized controlled studies are needed to confirm the benefits of CAM on adult cancer pain.

## 1. Introduction

Cancer rates are increasing globally. According to the World Health Organization (WHO) statistics, there were about 12.7 million cancer cases in 2008, and this number is expected to increase to 21 million by 2030. Cancer is the leading cause of death worldwide, accounting for 7.6 million deaths (around 13% of all deaths) in 2008. Pain is a common and burdensome symptom associated with cancer and its treatment [1, 2]. Most of cancer patients suffered pain due to the cancer itself (the tumor pressed on bones, nerves, or other organs), the treatment, or the tests done to diagnose cancer. It was said that 75%–90% cancer patients experienced pain during their illness and up to 50% of cancer pain is undertreated. It was reported that one quarter of the patients had newly diagnosed malignancies, one third of the patients are undergoing treatment, and three quarters of the patients with advanced disease experienced pain [3]. For those patients with metastasis to the other places, pain is especially prevalent. And it was reported that up to 80% of the cancer patients who have bone metastasis experienced pain [3].

Pain management is important in oncologic care and essential for maximizing patient outcomes [2, 4]. Mounting evidence showed that unrelieved pain significantly comprised overall quality of life and effective pain control was associated with survival [2, 4]. Health care practitioners depend heavily on opioid therapies for cancer pain. Although this therapy is very effective, it is with a lot of side effects, such as constipation, urinary retention, nausea, sedation, respiratory depression, myoclonus, delirium, sexual dysfunction, and hyperalgesia [5], so Complementary and Alternative Medicine (CAM), which is noninvasive and generally considered to be relatively free of toxicity, is used as an adjunct therapy with standard pain management techniques [6]. The earliest systematic review which included 18 trials showed that hypnosis, imagery, support groups, acupuncture, and healing touch were promising, particularly in the short term, but none can be recommended because of a paucity of rigorous trials [7]. And another review showed that approaches such as acupuncture, massage therapy, mind-body interventions, and music therapy could effectively reduce pain and enhance quality of life [6].

Now with the more and more published systematic reviews of CAM on adult cancer pain, it is necessary to use the methods of overview of systematic review to summarize the available evidence, appraise the evidence level, and give suggestions to future research and practice.

## 2. Methods

### 2.1. Inclusion and Exclusion Criteria

Only systematic reviews or meta-analyses of CAM on adult cancer pain were included. Patients were diagnosed with cancer, regardless of cancer types. The interventions were CAM or CAM in combination with conventional cancer treatments. Here, we used the definitions by the WHO: “A comprehensive term used to refer to both traditional medical systems such as traditional Chinese medicine, Indian ayurverda, Arabic unani medicine, and to various forms of indigenous medicine” [8]. The treatments include psychological and self-help therapies, physical therapies (aromatherapy, acupuncture, massage, reflexology, and shiatsu), and unconventional medicine or drugs (homeopathy, herbal medicine, Essiac, and Bach flower remedies) [7]. If there were several systematic reviews that evaluated the same interventions on adult cancer pain, we included the one that included most primary studies.

### 2.2. Data Source and Study Selection

We searched the Cochrane Library, PubMed, Embase, and ISI Web of Knowledge using the search term (Alternative medicine OR Homeopathy OR Acupuncture OR Reflexology OR Mind-body medicine OR Hypnosis OR Imagery OR Relaxation techniques OR Support groups OR Creative outlets OR music OR Biologic-based therapies OR Dietary supplements OR herbal OR nonherbal OR Manipulative and body-based methods OR Massage OR aromatherapy OR Magnet OR laser therapy OR Energy therapies OR Healing touch OR Reiki OR Complementary medicine OR Complementary Therapies OR Complementary Therapy OR Essiac OR traditional medicine OR shiatsu OR Ayurveda OR Phytotherapy) AND (cancers OR cancer OR neoplasm OR neoplasms OR tumor OR tumors OR adenocarcinoma) AND (pain OR pains) AND (meta-analysis OR meta-analyses OR systematic reviews OR systematic review) as title, abstract, or keyword. If possible, the medical heading terms such as MESH and EMTREE words were used. The reference lists of included systematic reviews were checked. All searches were conducted at 31 May, 2013, and updated at 31 August, 2013, and 17 February, 2014. There were not any restrictions in language, publication date, or publication type.

Two authors (Yanju Bao & Xiangying Kong) independently selected studies according to the inclusion criteria, and differences were resolved by a third reviewer (Baojin Hua).

### 2.3. Data Collection and Analysis

We used “assessment of multiple systematic reviews” (AMSTAR) [9] to assess the methodological quality of systematic reviews, as studies showed that it has satisfactory interobserver agreement, reliability, and construct validity [10, 11]. This checklist contains 11 items: “a priori” design, duplicate study selection and data extraction, comprehensive literature search, the status of publication used as an inclusion criterion, a list of studies (included and excluded), the characteristics of the included studies, assessing and documenting the scientific quality, using the scientific quality appropriately in formulating conclusions, appropriate methods to combine the findings, assessing the likelihood of publication bias, and the conflict of interest. This checklist could not give a total score for the methodological quality, so we adopted the revision version [12]: Revised Assessment of Multiple Systematic Reviews (R-AMSTAR). This R-AMSTAR did not destroy the content and construct validity of AMSTAR [12]. According to this checklist, 44 is the maximum value. However, for item 2 in R-AMSTAR, it focuses on data extracting, and it seemed to ignore study selection. As we know, efforts to enhance objectivity and avoid mistakes in study selection are important [13]. Thus, we added item 2.1 (duplicate study selection) according to item 2 in R-AMSTER. So the total score for R-AMSTER was 48. And we defined the systematic review as of high quality (the score >36), moderate quality (the score >24), low quality (the score >12), and very low quality (the score ≦12).

For the evaluation of the evidence levels for the outcomes, we used the Grading of Recommendations Assessment, Development and Evaluation (GRADE) approach which specifies four levels of quality: high, moderate, low, and very low quality evidence [14]. Two investigators (Liping Yang & Rui Liu) extracted data from included studies; differences were resolved by a third reviewer (Baojin Hua). The data extraction form summarized key characteristics of systematic reviews, including information on participants, interventions, outcomes, author's conclusions, and items about quality.

## 3. Results

### 3.1. Search Results

We found 1318 citations by searching medical databases (Cochrane library: 531, Pubmed: 237, Embase: 328, ISI web of knowledge: 222) and 42 citations by reference tracking. After screening titles and abstracts, we excluded duplications (236 citations), studies that were not about cancer (293 citations) or CAM (351 citations) and that were not systematic reviews (301 citations). We also excluded studies that were not about pain (75 citations) or cancer (69 citations) based on screening the full text. Finally, we included 35 papers [7, 15–48] for this overview, but we only reviewed 27 papers [19–21, 23–34, 36–40, 42–48] in our paper, as some of them were overlapped.

### 3.2. Characteristics of Included Systematic Reviews

One systematic review [7] was about all CAM interventions; it focused on acupuncture, music, herbal supplement/Ai-TongPing, massage, and healing touch. The other 34 systematic reviews were about psychosocial interventions [15, 16, 22, 38, 42, 44, 45], massage therapy [17, 21, 24, 33, 46], acupuncture [18, 35, 36, 41, 43], reflexology [23, 30, 32], Chinese herbal medicine [20, 40, 48], music therapy [31, 39], transcutaneous electric nerve stimulation [37], cupping [34], cannabis [27], lycopene [25],* Viscum album* L (European Mistletoe) [29], Reiki [28], homeopathic therapy (Traumeel) [19], creative arts therapies [47], and internal qigong [26].

### 3.3. Quality of Included Systematic Reviews

The total score for all systematic reviews ranges from 20 to 34 and all were of low or moderate quality. Of these systematic reviews, 23 [17, 18, 21, 22, 26–28, 30–33, 35–40, 42, 44–48] were of moderate quality and twelve [7, 15, 16, 19, 20, 23–25, 29, 34, 41, 43] were of low quality.

Six systematic reviews [7, 17, 31, 35, 37, 45] have “a priori” design, four systematic reviews [26, 31, 36, 39] conducted duplicate data extraction, six systematic reviews [17, 22, 38, 40, 43, 45] conducted duplicate study selection, 25 systematic reviews [7, 15–18, 21, 23–26, 29–35, 37, 40, 42–47] have comprehensive literature search, all systematic reviews provided a list of studies (included and excluded), 24 systematic reviews [15–17, 19–21, 24, 26–28, 30–32, 35–37, 39, 40, 42–46, 48] provided the characteristics of the included studies, and five systematic reviews [27, 30, 31, 33, 39] assessed and documented the scientific quality of the included studies. The other details of quality were presented in Table 1.

### 3.4. Summary of Findings

Five systematic reviews [17, 21, 24, 33, 46] were about massage on cancer pain. Two systematic reviews [17, 21] used the same included studies, and two systematic reviews [24, 33] included different studies, although they both conducted search after 2006. And the fifth [46] was a meta-analysis about breast cancer. So we reviewed four of them [21, 24, 33, 46]. Three of them [21, 24, 33] gave a conclusion that massage may have a beneficial effect on cancer pain without pooling the data. However, they included different primary studies: four randomized controlled trials (RCTs) [49–52] for systematic review by Wilkinson et al. [21], five RCTs [21, 50, 53–55] for systematic review by Ernst et al. [24], and three RCTs [51, 53, 56] for systematic review by Falkensteiner et al. [33]. The fifth systematic review [46] pooled the data and showed no benefits of massage on pain for breast cancer patients based on four different RCTs [57–60]. So based on available evidence, we could see that the conclusions for the benefits of massage on cancer pain were conflicted.

Four systematic reviews [18, 35, 36, 43] assessed the effects of acupuncture on cancer pain. The latest one [43] reviewed all available evidence of acupuncture plus drug therapy versus drug therapy on cancer pain and showed that acupuncture plus drug therapy might be better than drug therapy. Among the remaining three systematic reviews, the one by Choi et al. [36] was the most comprehensive one. This systematic review showed that acupuncture did not generate better effects on pain relief than drug therapy and sham acupuncture and that acupuncture plus drug therapy was better in managing cancer pain than drug therapy. In a word, available evidence showed that acupuncture plus drug therapy might be better than drug therapy in managing cancer pain.

Three systematic reviews [15, 16, 38] assessed the effects of psychosocial interventions on cancer pain and the latest [38] was the most comprehensive one. This systematic review [38] included 37 RCTs and showed that psychosocial interventions had medium-size effects on cancer pain severity and interference. Two systematic reviews [42, 45] assessed the effects of psychosocial interventions on pain for breast cancer patients. Both showed benefits on cancer pain, although they included different studies. Two systematic reviews [22, 44] assessed the effects of educational interventions on cancer pain, of them one [44] is the more comprehensive which showed that educational interventions can result in modest benefits in the management of cancer pain. Totally, psychoeducational interventions including psychosocial and educational interventions could be helpful for managing cancer pain.

Three systematic reviews [23, 30, 32] assessed the effects of reflexology on cancer pain. Two systematic reviews [23, 32] included one crossover RCT about breast and lung cancer, and the other systematic review [30] included two N-RCTs about breast cancer. Although these three systematic reviews included few studies, they all showed that reflexology may be beneficial in reducing cancer pain.

Three systematic reviews evaluated the effects of Chinese herbal medicine on cancer pain [20, 40, 48]. Systematic review by Xu et al. [20] showed that Chinese herbal medicine may be useful for managing cancer pain. The meta-analysis by Wang et al. [48] showed that Chinese herbal medicine plus conventional treatment increased the pain-relief rate as compared with the conventional treatment for pain secondary to bone metastases, and the meta-analysis by Bao et al. [40] showed that Chinese medicine, compound kushen injection, was associated with improving pain relief for bone cancer pain. Totally, we could see that Chinese herbal medicine may be beneficial in managing cancer pain.

Two systematic reviews [31, 39] evaluated the effects of music interventions on cancer pain. However, their included studies were different, as they were conducted at different times and/or by authors in different countries. These two systematic reviews showed that music interventions were associated with a moderate pain-reducing effect for cancer patients.

For other CAM interventions, lycopene [25], qigong [26], cupping [34], cannabis [27], Reiki [28], homeopathic therapy (Traumeel) [19], transcutaneous electric nerve stimulation (TENS) [37], creative arts therapies [47], and* Viscum album* L [29], there was only one systematic review for each. Studies showed that lycopene, qigong, cupping, cannabis, homeopathy (Traumeel), creative arts therapies, and Reiki might have beneficial effects on cancer pain. For TENS and* Viscum album* L, evidence was less consistently.

### 3.5. Evidence Level

Among the 27 systematic reviews we summarized, 18 systematic reviews [19–21, 23, 25, 27, 28, 31–34, 36, 37, 39, 40, 45, 46, 48] have small sample size, there were high heterogeneity in eight systematic reviews [19, 29, 31, 36, 39, 43, 46, 47], the risks of bias of 22 systematic reviews [19–21, 23–25, 27–29, 32, 34, 36–38, 40, 42–48] were high, and two systematic reviews [26, 30] included observational studies. So based on GRADE approach, the evidence levels for music, reflexology, lycopene, qigong, cupping, cannabis, Reiki, TENS, Chinese herbal medicine, homeopathy (Traumeel), creative arts therapies, and* Viscum album* L were low. For acupuncture, evidence was low for acupuncture or acupuncture + drug therapy (versus drug therapy) and very low for acupuncture (versus sham acupuncture). For massage therapy and psychoeducational interventions (psychosocial and educational interventions), the number of included studies was different, so the evidence level was low or moderate.

## 4. Discussion 

### 4.1. Summary of Finding

Based on available evidence, we could find that psychoeducational interventions, music interventions, acupuncture plus drug therapy, Chinese herbal medicine plus cancer therapy, compound kushen injection, reflexology, lycopene, TENS, qigong, cupping, cannabis, Reiki, homeopathy (Traumeel), and creative arts therapies might have beneficial effects on adult cancer pain. No benefits were found for acupuncture (versus drug therapy or shame acupuncture), and the results were inconsistent among studies for massage therapy, transcutaneous electric nerve stimulation (TENS), and* Viscum album* L plus cancer treatment. The methodological quality for primary studies was not very good and the evidence levels for these interventions were low or moderate, so firm conclusions could not be drawn. Based on all evidence we collected, we could not recommend any CAM interventions for adult cancer pain because of small sample size, high heterogeneity across studies, and high risk of bias for primary studies.

It was reported that the use of CAM among cancer patients is widespread and appears to be increasing [8]. Surveys on the use of CAM among cancer patients have been reported as high as 64% and as low as 7% [61]. For example, a survey on the use of CAM among patients with haematological cancers in 14 European countries showed that 36% of cancer patients in Europe have used one or more forms of CAM modalities [62]. Similar studies in New Zealand and Canada showed 42% and 43% prevalence rate of CAM use among cancer patients [63, 64].

Cancer patients were hoping to better control cancer and cancer-related pain, so they turned to CAM. Ernst [65] grouped the reasons given by patients for their use of CAM into push factors (negative) which pushes patients away from conventional medicine and pull factors (positive) which relates to the positive aspects of CAM. Study also showed that CAM might give cancer patients strength to go through the conventional therapies, relieve their symptoms, improve their quality of life [66, 67], and further increase the body's ability to fight off the disease [62].

Although more and more cancer patients turned to CAM to cure their disease, the evidence levels for the benefits of CAM on cancer pain were not satisfied. We could see from Table 2 that the evidence levels for all interventions were low or moderate. For most interventions, they were of low evidence level. Among these interventions, acupuncture plus drug therapy, Chinese herbal medicine, creative arts therapies, cannabis, cupping, lycopene, Reiki, qigong, music interventions, homeopathy (Traumeel), and reflexology might be beneficial in reducing cancer pain. Only two interventions (psychosocial intervention and massage therapy) from five systematic reviews were of moderate evidence level. However, definitive conclusions were not achieved for most of them due to the methodological problems and/or small sample size.

Where does the unsatisfied evidence level come from? According to 27 systematic reviews we summarized, small sample size, high heterogeneity across studies, and high risk of bias for primary studies were the reasons. Trials of complementary therapies often have relevant methodological weaknesses [68]. According to the systematic review by Garcia et al. [41], 33 of 41 RCTs about acupuncture for symptom management in cancer care were of high risk of bias. The earliest systematic review [7] of CAM in relieving cancer pain also found that the included RCTs were of high risk of bias. Studies have also found that insufficient sample size was common in CAM studies [69, 70]. For heterogeneity, that might be due to different administrative ways of CAM. So in the future, when designing the RCTs that compared CAM with placebo or other interventions, a rational sample size calculation should be well done. Meanwhile, the key methodological aspects, such as methods of randomization, concealed allocation, and blinding, should be well conducted and reported.

### 4.2. Strength and Limitations

Our overview was the first one which systematically reviewed available systematic reviews of CAM on adult cancer pain. We searched medical databases and hand-searched reference lists and used GRADE to evaluate the evidence levels for each kind of CAM. However, our systematic overview had its own limitations. First, we only included systematic reviews and this means that we did not include primary studies that evaluated CAM for adult cancer pain. For reflexology, the systematic reviews in our overview showed that reflexology might be beneficial on adult cancer pain, but a recent study showed no differences among reflexology, lay foot manipulation, and conventional care [71]. For yoga, there was not a systematic review that evaluated its effect on cancer pain. A recent RCT [72] which compared yoga with wait-list control showed that the yoga group reduced daily joint pain for breast cancer patients. Second, the critical problem for these primary RCTs of CAM on adult cancer pain was of low quality and of small sample size. For example, all included RCTs in the systematic review by Choi et al. [36] were associated with high risk of bias. Meanwhile, few studies were included in systematic reviews. For example, only one crossover RCT and two N-RCTs were about reflexology, two RCTs were about Reiki, and one RCT was about cupping.

### 4.3. Implications for Future Research and Practice

Due to high risk of bias for primary studies, the evidence levels for each CAM were low or moderate. So in the future, in order to prescribe CAM, the health care professionals should be more careful. Based on available evidence, we could not recommend any CAM interventions for cancer pain due to small sample size, high heterogeneity across studies, and high risk of bias for primary studies.

The methodological quality for primary studies was low and their sample size was small, so in the future large and well-designed RCTs should be conducted to confirm the conclusions of available systematic reviews.

## 5. Conclusions

Based on available evidence, psychoeducational interventions, music interventions, acupuncture plus drug therapy, Chinese herbal medicine plus cancer therapy, compound kushen injection, reflexology, lycopene, TENS, qigong, cupping, cannabis, Reiki, homeopathy (Traumeel), and creative arts therapies might have beneficial effects on cancer pain. No benefits were found for acupuncture (versus drug therapy or shame acupuncture), and the results were inconsistent among studies for massage therapy, transcutaneous electric nerve stimulation (TENS), and* Viscum album* L plus cancer treatment. The methodological quality for primary studies was not high and the evidence levels for these interventions were low or moderate, so firm conclusions could not be drawn. Based on all evidence we collected, we could not recommend any CAM interventions for adult cancer pain because of small sample size, high heterogeneity across studies, and high risk of bias for primary studies.

## Figures and Tables

**Table 1:** Quality of all included systematic reviews.

Study	1	2	2.1	3	4	5	6	7	8	9	10	11	Total	Quality
Bardia et al. 2006 [7]	ABC	A	0	ABCD	0	AD	AC	AB	A	0	0	B	24	Low
Bradt et al. 2011 [31]	ABC	ABC	0	ABCDE	0	ABCD	ABC	ABCD	A	C	A	AB	32	Moderate
Choi et al. 2012 [36]	BC	ABC	0	ABC	BD	AD	ABC	AB	A	BCD	0	AB	30	Moderate
Devine and Westlake 1995 [15]	BC	0	0	ABCD	0	AD	ABC	AB	A	A	0	A	23	Low
Devine 2003 [16]	BC	0	0	ABCD	0	AD	ABC	AB	A	A	0	A	23	Low
Ernst 2009 [23]	C	0	0	ABCD	0	AD	AC	AB	A	0	0	B	21	Low
Ernst 2009 [24]	BC	0	0	ABCD	AD	AD	ABC	AB	A	0	0	0	24	Low
Ernst et al. 2011 [32]	BC	0	0	ABCD	B	AD	ABC	AB	A	0	0	AB	25	Moderate
Falkensteiner et al. 2011 [33]	BC	0	A	ABDE	B	ABCD	AC	ABCD	A	0	0	0	27	Moderate
Fellowes et al. 2004 [17]	ABC	0	ABC	ABCDE	AD	ABCD	ABC	AB	0	0	0	B	31	Moderate
Haseen et al. 2009 [25]	BC	A	A	ABCD	0	AD	AC	AB	A	0	0	B	24	Low
Hurlow et al. 2012 [37]	ABC	A	0	ABCD	0	ABCD	ABC	AB	A	C	0	AB	27	Moderate
Garcia et al. 2013 [41]	BC	A	A	ABC	B	AD	0	AB	0	0	0	B	22	Low
Kienle and Kiene 2010 [29]	BC	0	0	ABCD	0	AD	AC	AB	A	0	0	A	22	Low
Kim et al. 2010 [30]	BC	0	0	ABCD	D	AD	ABC	ABCD	A	0	0	AB	27	Moderate
Lee et al. 2005 [18]	BC	A	0	ABCD	D	ABCD	AC	AB	A	0	0	A	26	Moderate
Lee et al. 2009 [26]	BC	ABC	0	ABCD	0	ABCD	ABC	AB	A	0	0	A	27	Moderate
Lee et al. 2011 [34]	BC	A	0	ABCD	D	AD	AC	AB	A	0	0	A	24	Low
Martín-Sánchez et al. 2009 [27]	BC	0	0	ABC	AD	AD	ABC	ABCD	0	C	AB	AB	29	Moderate
Paley et al. 2011 [35]	ABC	0	0	ABCD	AD	ABCD	ABC	AB	A	BCD	AB	B	31	Moderate
Gorin et al. 2012 [38]	BC	0	ABC	ABC	B	ABCD	0	0	0	BC	AB	B	27	Moderate
Edwards et al. 2004 [45]	ABC	A	ABC	ABCD	B	ABCD	ABC	AB	AB	BCD	0	A	34	Moderate
Johannsen et al. 2013 [42]	BC	0	AB	ABCD	B	AD	ABC	AB	AB	BCD	0	A	28	Moderate
Bennett et al. 2009 [22]	BC	0	ABC	ABD	0	AD	A	AB	AB	BCD	0	A	26	Moderate
Marie et al. 2013 [44]	BC	0	A	ABCD	B	AD	ABC	AB	AB	BCD	0	0	27	Moderate
Vandervaart et al. 2009 [28]	BC	0	0	ABC	0	AD	ABC	AB	A	0	0	A	22	Moderate
Wilkinson et al. 2008 [21]	BC	0	A	ABCDE	AD	ABCD	ABC	AB	A	0	0	B	28	Moderate
Xu et al. 2007 [20]	B	0	0	ABC	0	AD	ABC	AB	A	0	0	0	20	Low
Wang et al. 2013 [48]	BC	0	0	ABD	AC	AD	ABC	AB	A	BCD	ABC	A	28	Moderate
Bao et al. 2013 [40]	BC	A	ABC	ABCD	0	AD	ABC	AB	A	BCD	AB	A	31	Moderate
Zhang et al. 2012 [39]	BC	ABC	0	ABC	0	AD	ABC	ABCD	A	C	0	B	27	Moderate
Pan et al. 2013 [46]	BC	0	0	ABCD	B	AD	ABC	AB	AB	BCD	0	B	27	Moderate
Lian et al. 2013 [43]	BC	0	ABC	ABCD	0	AD	ABC	A	0	0	0	0	24	Low
Milazzo et al. 2006 [19]	BC	0	0	ABC	0	AD	ABC	AB	A	0	0	B	22	Low
Puetz et al. 2013 [47]	BC	0	0	ABCD	B	AD	0	AB	A	BCD	ABC	B	26	Moderate

**Table 2:** Summary of finding for 27 included systematic reviews.

Study	Search time	Study (sample size)	Interventions	Comparison	Main result	Recommendation	Evidence level
Bradt et al. 2011 [31]	September 2010	7 RCTs (391)	Music	Standard care	Benefits	Yes	Low^1,2^
Choi et al. 2012 [36]	April 2011	15 RCTs (1157)	Acupuncture	Drug	No effect	No	Low^2,3^
			Acupuncture + drug	Drug	Benefits		Low^2,3^
			Acupuncture	Sham acupuncture	No effect		Verylow^1,2,3^
Lian et al. 2013 [43]	June 2010	6 RCTs (476)	Acupuncture + drug	Drug	Benefits	No	Low^2,3^
Ernst 2009 [23] and Ernst et al. 2011 [32]^*※*^	September 2010	1 RCT (23)	Reflexology	No treatment	Benefits	No	Low^1,3^
Ernst 2009 [24]	November 2008	5 RCTs (781)	Massage	Control	Benefits	No	Moderate^3^
Falkensteiner et al. 2011 [33]	July 2010	3 RCTs, 2 N-RCT (1841)	Massage	Control	Benefits	No	Moderate^1^
Pan et al. 2013 [46]^§^	November 2012	4 RCTs	Massage	Control	No effect	No	Very low^1,2,3^
Wilkinson et al. 2008 [21]	2006	4 RCTs (212)	Massage	Control	Benefits	No	Low^1,3^
Haseen et al. 2009 [25]^#^	January 2009	1 RCT (54)	Lycopene	No treatment	Benefits	No	Low^1,3^
Hurlow et al. 2012 [37]	April 2008	3 RCTs (88)	TENS	Placebo	Inconsistent	No	Low^1,3^
Kienle and Kiene 2010 [29]	October 2009	5 RCTs (745)	*Viscum album* l + cancer treatment	Cancer treatment	Inconsistent	No	Low^2,3^
Kim et al. 2010 [30]^§^	July 2010	2 N-RCTs (58)	Reflexology	No treatment	Benefits	No	Low^4^
Lee et al. 2009 [26]^§^	February 2009	1 CCT (133)	Qigong	Control	Benefits	No	Low^4^
Lee et al. 2011 [34]	January 2009	1 RCT (60)	Cupping	Drug	Benefits	No	Low^1,3^
Martín-Sánchez et al. 2009 [27]	February 2008	5 RCTs (290)	Cannabis	Placebo	Benefits	No	Low^1,3^
Gorin et al. 2012 [38]	2010	37 RCTs (4199)	Psychosocial intervention	Usual care	Benefits	Yes	Moderate^3^
Edwards et al. 2004 [45]^§^	June 2011	3 RCTs (279)	Psychosocial intervention	Usual care	Benefits	Yes	Low^1,3^
Johannsen et al. 2013 [42]^§^	December 2012	26 trials (1786)	Psychosocial intervention	Usual care	Benefits	Yes	Moderate^3^
Marie et al. 2013 [44]	May 2012	15 trials (1710)	Education	Usual care	Benefits	Yes	Moderate^3^
Vandervaart et al. 2009 [28]	December 2008	2 RCTs (40)	Reiki	Control	Benefits	No	Low^1,3^
Xu et al. 2007 [20]	2006	41 RCTs (2902)	Chinese herbal medicine + cancer therapy	Cancer therapy	Benefits	No	Low^1,3^
Wang et al. 2013 [48]	December 2012	16 RCTs (1008)	Chinese herbal medicine + conventional treatment	Conventional treatment	Benefits	No	Low^1,3^
Bao et al. 2013 [40]	December 2012	7 RCTs (521)	Compound kushen injection	Radiotherapy or bisphosphonates	Benefits	No	Low^1,3^
Zhang et al. 2012 [39]	March 2011	7 RCTs (603)	Music intervention	Standard care	Benefits	No	Low^1,2^
Milazzo et al. 2006 [19]	2005	2 RCTs (57)	Homeopathy (Traumeel)	No treatment/Placebo	Benefits	No	Low^1,3^
Puetz et al. 2013 [47]	January 2012	18 trials (1237)	Creative arts therapies	No treatment	Benefits	No	Low^2,3^

TENS: Transcutaneous Electric Nerve Stimulation; RCT: randomized controlled trial; N-RCT: non-randomized controlled trial; CCT: clinical controlled trial; ^*※*^breast cancer and lung cancer; ^§^Breast cancer; ^#^Prostate cancer; ^1^the total sample size was small; ^2^there was a high heterogeneity; ^3^the risk of bias was high; ^4^observational study.
